# New data on karyotypes of lace bugs (Tingidae, Cimicomorpha, Hemiptera) with analysis of the 18S rDNA clusters distribution

**DOI:** 10.3897/CompCytogen.v12i4.30431

**Published:** 2018-12-13

**Authors:** Natalia V. Golub, Viktor B. Golub, Valentina G. Kuznetsova

**Affiliations:** 1 Zoological Institute, Russian Academy of Sciences, Universitetskaya nab. 1, St. Petersburg 199034, Russia Zoological Institute, Russian Academy of Sciences St. Petersburg Russia; 2 Voronezh State University, Universitetskaya pl. 1, Voronezh, 394006, Russia Voronezh State University Voronezh Russia

**Keywords:** Karyotype, chromosome number, sex chromosomes, FISH, rDNA, (TTAGG)*_n_*, lace bugs, Tingidae, Heteroptera

## Abstract

The karyotypes of 10 species from 9 genera of the family Tingidae (Hemiptera, Heteroptera, Cimicomorpha) are described and illustrated for the first time. These species are: *Agrammaatricapillum* (Spinola, 1837), *Catoplatuscarthusianus* (Goeze, 1778), *Dictylaplatyoma* (Fieber, 1861), *Lasiacanthahermani* Vásárhelyi, 1977, *Oncochilasimplex* (Herrich-Schaeffer, 1830), Tingis (Neolasiotropis) pilosa Hummel, 1825, and T. (Tropidocheila) reticulata Herrich-Schaeffer, 1835, all with 2n = 12A + XY, as well as *Acalyptamarginata* (Wolff, 1804), Derephysia (Paraderephysia) longispina Golub, 1974, and *Dictyonotastrichnocera* Fieber, 1844, all with 2n = 12A + X(0). Moreover, genera *Catoplatus* Spinola, 1837, *Derephysia* Spinola, 1837, and *Oncochila* (Herrich-Schaeffer, 1830) were explored cytogenetically for the first time. Much as all other hitherto studied lace bugs, the species studied here have 12 autosomes but differ in their sex chromosome systems. The ribosomal clusters were localized on male meiotic cells of all ten species already mentioned and, additionally, in *Acalyptacarinata* Panzer, 1806 known to have 2n = 12A + X (Grozeva and Nokkala 2001) by fluorescence *in situ* hybridization (FISH) using a PCR amplified 18S rDNA fragment as a probe. In all cases, rDNA loci were located interstitially on a pair of autosomes. Furthermore, two species possessed some additional rDNA clusters. Thus, *Acalyptamarginata* showed clearly defined interstitial clusters on one more pair of autosomes, whereas *Derephysialongispina* had a terminal cluster on the X-chromosome. FISH performed with the telomeric (TTAGG)*_n_* probe did not reveal labelling in chromosomes of any species studied. Hence, the results obtained provide additional evidence for the karyotype conservatism, at least regarding the number of autosomes, for variation in chromosomal distribution of rDNA loci between species and for the lack of the ancestral insect telomeric sequence TTAGG in lace bugs. Preliminary taxonomic comments are made basing on some cytogenetic evidence.

## Introduction

Tingidae (lace bugs) are a relatively large family belonging to one of the evolutionarily advanced true bug infraorders Cimicomorpha. The family comprises approximately 2600 species and more than 270 genera in the two currently recognized subfamilies, the Tinginae and the Cantacaderinae (Golub and Popov 2016). The currently available cytogenetic evidence is confined to the largest and most diverse subfamily Tinginae (Grozeva and Nokkala 2001, Golub et al. 2015, 2016, 2017, for other references see Ueshima 1979). Based on the present knowledge, the subfamily exhibits karyotype conservatism, at least in terms of the number of autosomes which is 12 in all hitherto studied species. On the other hand, the species can differ in sex chromosome systems which are of either an XY or an X(0) type, the former being clearly more characteristic of lace bugs. By now, 38 species from 18 genera have been karyotyped and the great majority of these species, 34 in 16 genera, were shown to have 2n = 14 (12A + XY) in males.

In recent years, cytogenetic studies with the use of fluorescence *in situ* hybridization (FISH) have advanced our understanding the karyotype structure of lace bugs (Golub et al. 2015, 2016, 2017). It became evident that, despite very similar karyotypes, these insects show significant interspecific differences in the major rDNA loci distribution. The 18S rDNA sites can appear either on sex chromosomes or on autosomes being in turn located either interstitially or terminally on a chromosome. Likewise, our studies suggest that lace bugs lack the insect-type telomeric sequence TTAGG (Golub et al. 2015, 2017).

To further explore the karyotype structure and evolution in lace bugs, we examined distribution of the rRNA gene loci in eleven additional species including *Acalyptacarinata* (Panzer, 1806), *A.marginata* (Wolff, 1804), *Agrammaatricapillum* (Spinola, 1837), *Catoplatuscarthusianus* (Goeze, 1778), Derephysia (Paraderephysia) longispina Golub, 1974, *Dictyonotastrichnocera* Fieber, 1844, *Dictylaplatyoma* (Fieber, 1861), *Lasiacanthahermani* Vásárhelyi, 1977, *Oncochilasimplex* (Herrich-Schaeffer, 1830), Tingis (Neolasiotropis) pilosa Hummel, 1825, and T. (Tropidocheila) reticulata Herrich-Schaeffer, 1835. In each species, we mapped the insect-type telomere motif (TTAGG)*_n_*. All species (besides *A.carinata*) as well as the genera *Catoplatus* Spinola, 1837, *Derephysia* Spinola, 1837, and *Oncochila* Stål, 1873 were studied here for the first time in terms of standard chromosome complement.

## Material and methods

Specimens of 11 lace bug species from 9 genera were sampled from the Voronezh and Astrakhan provinces of Russia (Table 1). Species identification was made by V. Golub. Only male specimens were used. Males were fixed in 3:1 fixative (96% ethanol: glacial acetic acid) and stored at 4 °C. Chromosomal preparations were obtained from the testes and made permanent using a dry ice quick-freezing technique. For standard karyotype analysis, a Feulgen-Giemsa method developed by Grozeva and Nokkala (1996) was used. FISH with 18S rDNA- and (TTAGG)*_n_*-telomeric probes was carried out according to Grozeva et al. (2010). In brief, the probes were simultaneously used in double FISH experiments. Telomeric sequences and 18S rDNA probes were labelled by PCR with Rhodamine-5-dUTP (GeneCraft, Köln, Germany) and Biotin-16-dUTP, respectively. The probe for 18S rDNA was detected by NeutrAvidin fluorescein conjugate (Invitrogen, Karlsbad, CA, USA). Chromosomes were counterstained with DAPI (Sigma-Aldrich). As a positive control for the efficacy of our (TTAGG)*_n_*FISH experiments, we used chromosome preparations from the jumping plant bug species (Hemiptera, Psylloidea) known to be (TTAGG)*_n_* – positive (Maryańska-Nadachowska et al. 2018).

**Table 1.** Material used for chromosome analysis.

Species	Data and place of collection	Number of males examined	Number of nuclei studied by	
			routine staining	FISH
1. *Acalyptacarinata*	30.04.2017, Voronezh Province, Russia	1	23	12
2. *Acalyptamarginata*	30.4 – 05.05.2017, Voronezh Province, Russia	12	28	24
3. *Agrammaatricapillum*	01.06.2017, Bogdinsko-Baskunchakski Nature Reserve, Astrakhan Province, Russia	2	–	17
4. *Catoplatuscarthusianus*	31.07.2017, Voronezh Province, Russia	20	65	47
5. Derephysia (Paraderephysia) longispina	7.06.2017, Voronezh Province, Russia	22	31	45
6. *Dictylaplatyoma*	29 – 31.05.2017, Bogdinsko-Baskunchakski Nature Reserve, Astrakhan Province, Russia	2	–	14
7. *Dictyonotastrichnocera*	20.06 – 01.07.2017, Voronezh Province, Russia	3	38	24
8. *Lasiacanthahermani*	2.06 – 16.06.2017, Voronezh Province, Russia	2	22	11
9. *Oncochilasimplex*	22.06 – 03.07.2017, Voronezh Province, Russia 27.07.2017, Lipetsk Province, Russia	7	32	23
10. Tingis (Tropidocheila) reticulata	20.06 – 4.07.2017, Voronezh Province, Russia	20	–	31
11. Tingis (Neolasiotropis) pilosa	8.06 – 25.06.2017 Voronezh Province, Russia	10	–	22

Chromosome slides were analyzed under a Leica DM 6000 B microscope (Leica Microsystems Wetzlar GmbH, Germany) with a 100× objective. Images were taken with a Leica DFC 345 FX camera using Leica Application Suite 3.7 software with an Image Overlay module.

All cytogenetic preparations and remains of the specimens from which the preparations were made are stored at the Zoological Institute of RAS, St. Petersburg.

## Results

### *Acalypta carinata*

**Figure 1.**
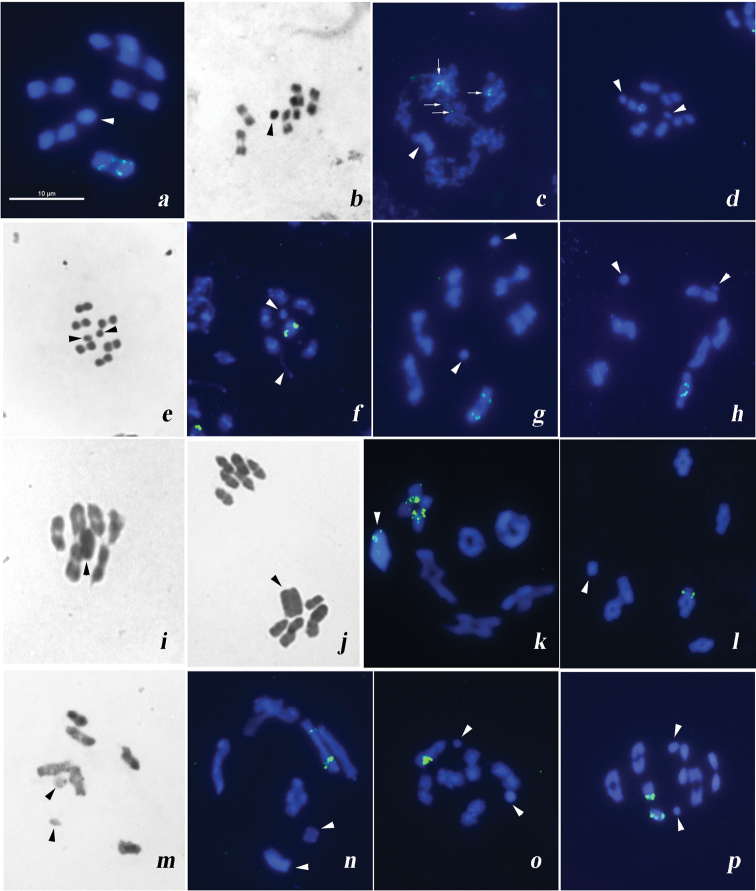
Male meiotic chromosomes of lace bug species after standard Schiff-Giemsa staining (**b, e, i, j, m**) and FISH with 18S rDNA and (TTAGG)_n_ telomeric probes (**a, c, d, f-h, k, l, n–p**). **a***Acalyptacarinata* metaphase I (MI) **b, c***Acalyptamarginata* MI (**b**) late prophase (**c**); 18S rDNA FISH signals on several bivalents are indicated by arrows **d***Agrammaatricapillum* MI **e, f***Catoplatuscarthusianus* MI (**e**) late prophase (**f**) **g***Lasiacanthahermani* prophase I/MI transition **h***Dictylaplatyoma* prophase I/MI transition **i, g, k**Derephysia (Paraderephysia) longispina MI (**i**) anaphase I (**j**) diakinesis (**k**) **l***Dictyonotastrichnocera* prometaphase I **m, n***Oncochilasimplex* prophase I to MI transition **o**Tingis (Tropidocheila) reticulata prometaphase I **p**Tingis (Neolasiotropis) pilosa. Sex chromosomes are indicated by arrowheads. Scale bar: 10 μm.

2n = 12A + X (Fig. 1a – FISH)

This species was previously karyotyped by Grozeva and Nokkala (2001), and our observations corroborate with their data. At spermatocyte metaphase I (MI), six bivalents of autosomes and a univalent X-chromosome are present (Fig. 1a: n = 6AA + X). Bivalents are more or less close in size, and the X is about half the size of the bivalents.

Numerous 18S rDNA FISH signals are located interstitially on both homologues of one of the autosome pairs. FISH with the pentamer (TTAGG)*_n_* as the probe did not label the telomeres in chromosomes of *A.carinata*.

### *Acalypta marginata*

2n = 12A + X (Fig. 1b – standard staining; Fig. 1c – FISH)

At spermatocyte MI, six bivalents of autosomes and a univalent X-chromosome are present (Fig. 1b: n = 6AA + X). Bivalents are of similar size, and the X is about half the size of the bivalents.

During late prophase, 18S rDNA FISH signals are visible on several bivalents being numerous and most pronounced on two of them (Fig. 1c). FISH with (TTAGG)*_n_* as the probe did not label the telomeres in chromosomes of *A.marginata*.

### *Agramma atricapillum*

2n = 12A + XY (Fig. 1d – FISH).

At early MI, six bivalents of autosomes and X and Y chromosomes as univalents are present (Fig. 1d: n = 6AA + X + Y). Bivalents are of similar size. Sex chromosomes are approximately similar in size and placed separately from each other at this stage – that is characteristic of the true bugs (Ueshima 1979).

18S rDNA FISH signals are located interstitially on one of the bivalents being clearer defined on one of its homologues. FISH with (TTAGG)*_n_* as the probe did not label the telomeres in chromosomes of *A.atricapillum*.

### *Catoplatus carthusianus*

2n = 12A + XY (Fig. 1e – standard staining; Fig. 1f – FISH).

At MI subjected to a routine staining as well as in a late prophase cell after FISH six bivalents of autosomes and univalent X and Y chromosomes are present (Fig. 1e, f: n = 6AA + X + Y). Bivalents are of similar size. Sex chromosomes are approximately similar in size and form a pseudo-bivalent at MI.

18S rDNA FISH revealed massive signals on one of the autosome pairs (Fig. f). FISH with (TTAGG)*_n_* as the probe did not label the telomeres in chromosomes of *C.carthusianus*.

### *Lasiacantha hermani*

2n = 12A + XY (Fig. 1g – FISH)

During the prophase I to MI transition, six bivalents of autosomes and univalent X and Y chromosomes are revealed (n = 6AA + X + Y). Bivalents are of similar size. Sex chromosomes are similar in size and placed separately from each other at this stage.

Bright 18S rDNA FISH signals are located interstitially on both homologues of one of the bivalents. FISH with (TTAGG)*_n_* as the probe did not label the telomeres in chromosomes of *L.hermani*.

### *Dictyla platyoma*

2n = 12A + XY (Fig. 1h – FISH)

During the prophase I to MI transition, six bivalents of autosomes and univalent X and Y chromosomes are revealed (n = 6AA + X + Y). Bivalents are of similar size. Sex chromosomes are similar in size and placed separately from each other at this stage.

Bright 18S rDNA FISH signals are located interstitially on both homologues of one of the bivalents. FISH with (TTAGG)*_n_* as the probe did not label the telomeres in chromosomes of *D.platyoma*.

### Derephysia (Paraderephysia) longispina

2n = 12A + X (Fig. 1i, j – standard staining; Fig. 1k – FISH)

At MI, six bivalents of autosomes and a univalent X-chromosome are present (Fig. 1i: 6AA + X). Bivalents are very large and of similar size. The X is the largest element of the set and appears positively heteropycnotic at this stage. It goes to one of the daughter nuclei (pre-reduction) at anaphase I (AI), resulting in different MII cells, respectively, that with 6 autosomes only and that with 6 autosomes plus X-chromosome, the latter being split into the chromatids (Fig. 1j).

Figure 1k shows a diakinesis after FISH with 18S rDNA probe demonstrating the presence of multiple signals on one of the bivalents as well as on the X. These FISH signals are interstitial on the bivalent while telomeric on the X. FISH with (TTAGG)*_n_* as the probe did not label the telomeres in chromosomes of *D.longispina*.

### *Dictyonota strichnocera*

2n = 12A + X (Fig. 1l – FISH)

The prometaphase I shows six bivalents of autosomes and a univalent X-chromosome (n = 6AA + X). Bivalents are of similar size, while the X is about half the size of the bivalents.

Bright 18S rDNA FISH signals are located interstitially on one of the bivalents, being however visible on one homologue only. FISH with (TTAGG)*_n_* as the probe did not label the telomeres in chromosomes of *D.strichnocera*.

### *Oncochila simplex*

2n = 12A + XY (Fig. 1m – standard staining; Fig. 1n – FISH)

During the prophase I to MI transition, six bivalents of autosomes and univalent X and Y chromosomes placed separately from each other are revealed (Fig. 1m, n: n = 6AA + X + Y). Bivalents are approximately similar in size, and the X is twice as large as the Y.

Signals of the 18S rDNA probe are located interstitially on both homologues of one of the bivalents being more massive and bright on one of them (Fig. 1n). FISH with (TTAGG)*_n_* as the probe did not label the telomeres in chromosomes of *O.simplex*.

### Tingis (Tropidocheila) reticulata

2n = 12A + XY (Fig. 1o – FISH)

Prometaphase I shows six bivalents of autosomes and X and Y chromosomes which are placed separately from each other at this stage. Bivalents are of similar size, and the X is twice as large as the Y (Fig. 1o).

Massive 18S rDNA FISH signals are located interstitially on one of the bivalents. FISH with (TTAGG)*_n_* as the probe did not label the telomeres in chromosomes of *T.reticulata*

### Tingis (Neolasiotropis) pilosa

2n = 12A + XY (Fig. 1p – FISH)

During the MI to AI transition, six bivalents of autosomes and a pseudo-bivalent formed by X and Y chromosomes are revealed (n = 6AA + XY). At this stage, bivalents appear as similar in size, while X -chromosome is twice as large as the Y (Fig. 1p).

One of the bivalents shows bright 18S rDNA signals, the signals locating most likely interstitially as seen on one homologue of this bivalent at least. FISH with (TTAGG)*_n_* as the probe did not label the telomeres in chromosomes of *T.pilosa*.

## Discussion

### Chromosome numbers and sex chromosome systems

For the first time, we studied the standard karyotypes of 10 lace bug species belonging to 9 genera of the subfamily Tinginae. Our data on chromosome numbers and sex chromosome systems of these species reinforce the statement (Ueshima 1979, Grozeva and Nokkala 2001, Golub et al. 2015, 2016, 2017) that lace bugs exhibit extraordinary stability of karyotypes in terms of the number of autosomes. Much as all previously studied species (38 species, 18 genera), all the species explored in the present study showed 12 autosomes in their diploid karyotypes suggesting thus that this number is under stabilizing natural selection. On the other hand, these species, despite the same autosome number, differ by sex chromosome systems which are of an X(0) type in 3 species (in genera *Derephysia, Acalypta* Westwood, 1840, and *Dictyonota* Curtis, 1827) and of an XY type in 8 species (in genera *Agramma* Stephens, 1829, *Catoplatus*, *Dictyla* Stål, 1874, *Lasiacantha* Stål, 1873, *Oncochila*, *Tingis* Fabricius, 1803) respectively. The predominance of the XY-system is typical for the family Tingidae as a whole, being found in 41 of the 48 hitherto studied species. Since more than 70% of the cytogenetically studied species of Heteroptera have the XY system and only about 14% possess the X(0) system, the former system is considered typical for this suborder as a whole (Papeschi and Bressa 2006).

In summary, based on the currently available evidence, the karyotype of 2n = 12A + XY/XX (male/female) can be taken as the modal one for the family Tingidae, at least for the subfamily Tinginae. Moreover, we like to suggest that the XY system is the ancestral one in lace bugs and the X(0) is secondary resulting from the loss of the Y chromosome (see also Nokkala and Nokkala 1984).

The distribution of the sex chromosome systems in Tingidae seems to allow some preliminary taxonomic speculations. All the seven X(0)- lace bug species belong to the phylogenetically close genera *Acalypta* (*A.parvula* Fallén, 1897, *A.carinata*, *A.nigrina* Fallén, 1897, *A.marginata*; Grozeva and Nokkala 2001, present paper), *Derephysia* (*D.longispina*; present paper), *Kalama* Puton, 1876 (*K.tricornis* Schrank, 1801; Grozeva and Nokkala 2001), and *Dictyonota* (*D.strichnocera*; present paper). On the other hand, according to Southwood and Leston (1959), *Acalyptaparvula* and another species of *Dictyonota* (*D.fuliginosa* Costa, 1853) both originating from British Islands have an XY system. However, neither illustrations nor descriptions of the karyotypes were provided in the above-mentioned publication, so the credibility of these data is questionable. It is of interest that all the above genera share, besides common X(0) system, some morphological similarities, including the absence of the cuticular frame (peritrema) of the metatoracic scent glands in adults and bucculae not closed anteriorly (Horváth 1906, Kerzhner and Jaczewski 1964, Péricart 1983). Thus, these cytogenetic and morphological characters can be considered as synapomorphies for the genera *Acalypta*, *Derephysia*, *Kalama*, and *Dictyonota*. Furthermore, these genera have almost exclusively Holarctic distribution (Drake and Ruhoff 1965, Golub 1975, Péricart 1983, Péricart and Golub 1996, Froeschner 2001).

### Karyotype structure

In the tingid karyotypes, autosomes are more or less close in size or, most probably, form gradually decreasing series in size (Grozeva and Nokkala 2001, Golub et al. 2015, 2016, 2017) and this is also true for the species used in the present study. Because of the uniform chromosome size and, additionally, of the holokinetic nature of chromosomes, it is almost impossible to identify separate chromosome pairs in a given karyotype when standard chromosome staining techniques are applied. Moreover, C-banding appeared to be not very helpful for the identification due to scarce and uniform C-patterns of the chromosomes although various species show some differences in the C-banding picture (Grozeva and Nokkala 2001). Sex chromosomes, both X and Y, are always small, smaller than any of autosomes of the set. The only so far known exception is *Derephysialongispina* from the present study. The karyotype of this species is unique in having rather large chromosomes, the X-chromosome being at least twice as large as any autosome. The observed differences may be of taxonomic significance. It would be of interest to compare the genome size in lace bug species with different chromosomal length. Furthermore, in *D.longispina* we were able to observe that the X-chromosome separated reductionally during first meiotic division (pre-reduction). The orthodox sex chromosomes pre-reduction seems to be characteristic of the Tingidae as a whole (Ueshima 1979, Grozeva and Nokkala 2001, present study). Interestingly, pre-reduction distinguishes lace bugs from all other Cimicomorpha families, for which sex chromosome post-reduction, i.e. the inverted sequence of sex chromosome divisions in male meiosis, is typical (Ueshima 1979).

### rDNA-FISH

All 11 species studied here by FISH with 18S rDNA probes showed major rRNA gene clusters on an autosome pair. Unfortunately, based on the present data we cannot conclude whether these species share a syntenic location of their rDNA arrays since the chromosome pairs are of similar size and morphology within karyotypes. In one species, *Derephysialongispina*, an additional rDNA site was revealed on the X-chromosome. Furthermore, *Acalyptamarginata* displayed several rDNA loci housed on two pairs of autosomes, at least. These two species represent two novel patterns of rDNA distribution in lace bugs. Thus, the following patterns are currently known in Tingidae: on the X-chromosome, on both X and Y chromosomes, on one or two pairs of autosomes, and both on the X and one pair of autosomes. A wide variety of rDNA location between species sharing the same chromosome number has also been reported in some other Cimicomorpha families (Severi-Aguiar and de Azeredo-Oliveira 2005, Morielle-Souza and Azeredo-Oliveira 2007, Bardella et al. 2010, Grozeva et al. 2011, 2015, Poggio et al. 2011, Pita et al. 2013, Panzera et al. 2012, 2014, 2015).

Noteworthy is an interstitial location of the rDNA sites discovered in all lace bug species from the present study, at least in terms of autosomal location. Such is the case in the majority of lace bugs studied so far (Golub et al. 2016, 2017) suggesting this localization to be most characteristic of Tingidae. On the other hand, a terminal rDNA location has frequently been reported in other families of Cimicomorpha, e.g. Reduviidae and Cimicidae (Poggio et al. 2011, 2014, Panzera et al. 2012, Bardella et al. 2014, Grozeva et al. 2010, 2014). It is worth noting however that evidence was usually based on MI plates which characteristically show highly condensed chromosomes and may thus result in a misinterpretation.

### (TTAGG)*_n_*-FISH

Like all previously studied lace bug species in the genera *Agramma*, *Catoplatus*, *Dictyla*, *Elasmotropis* Stål, 1874, *Galeatus* Curtis, 1833, and *Tingis* (Golub et al. 2015, 2017), all species used in the present study representing 4 further genera, namely, *Acalypta*, *Dictyonota*, *Lasiacantha*, and *Oncochila*, showed no labelling with the pentameric repeat (TTAGG)*_n_*. At the moment, all accumulated information on different insect groups supports the hypothesis suggested by Frydrychová et al. (2004) that the TTAGG telomeric repeat is ancestral one in the class Insecta. However, this repeat was either changed to another sequence (e.g. TCAGG in some beetles; Mravinac et al. 2011) or lost many times along various branches of the insect phylogenetic tree (Frydrychová et al. 2004), including some branches of Heteroptera. Within Heteroptera, the (TTAGG)*_n_* telomeric sequence is present in all hitherto studied basal families (Kuznetsova et al. 2012, Angus et al. 2017, Chirino et al. 2017) while was not found in all but one remaining families belonging to the evolutionarily advanced infraorders Cimicomorpha and Pentatomomorpha (Frydrychová et al. 2004, Grozeva et al. 2011, Golub et al. 2015, 2017, present paper). Specifically, the family Reduviidae (Cimicomorpha) is the only exception in this respect (Pita et al. 2016). The finding of the ancestral telomere motif (TTAGG)*_n_* in the youngest reduviid subfamily Triatominae (Pita et al. 2016) is of obvious interest and invites further investigation.

In sum, the data presented here add to the considerable body of previously published evidence that the lace bugs (1) are characterized by very conservative karyotypes with 12 autosomes and the XY as the most typical sex chromosome system, (2) lack the insect telomeric sequence TTAGG and (3) differ from each other in the location of the rRNA genes in their genomes. The results have identified *D.longispina* as the species with the largest X- chromosome in the family Tingidae. The comparative survey has also shown that the evolutionarily secondary sex chromosome system X(0) is restricted to the genera sharing some specific morphological characteristics and can be useful thus to clarify the phylogenetic relations between the lace bug higher taxa.
